# Carnitine palmitoyl transferase 1A is a novel diagnostic and predictive biomarker for breast cancer

**DOI:** 10.1186/s12885-021-08134-7

**Published:** 2021-04-15

**Authors:** Zheqiong Tan, Yaru Zou, Man Zhu, Zhenzhao Luo, Tangwei Wu, Chao Zheng, Aqing Xie, Hui Wang, Shiqiang Fang, Shuiyi Liu, Yong Li, Zhongxin Lu

**Affiliations:** 1 Department of Medical Laboratory, the Central Hospital of Wuhan, Tongji Medical College, Huazhong University of Science and Technology, Wuhan, 430014 Hubei China; 2 Department of Clinical Laboratory, Wusong Central Hospital, Baoshan District, Shanghai, 200940 China; 3 Cancer Research Institute of Wuhan, Wuhan, 430014 Hubei China; 4 Department of Central Laboratory, the Central Hospital of Wuhan, Tongji Medical College, Huazhong University of Science and Technology, Wuhan, 430014 Hubei China; 5 Department of Cancer Biology, Lerner Research Institute, Cleveland Clinic, Cleveland, OH 44195 USA; 6 Department of Medicine, Section of Epidemiology and Population Sciences, Baylor College of Medicine, Houston, TX 77030 USA

**Keywords:** Carnitine palmitoyl transferase 1A, Breast cancer, Biomarker, Diagnosis

## Abstract

**Background:**

Carnitine palmitoyl transferase 1A (CPT1A), the key regulator of fatty acid oxidation, contributes to tumor metastasis and therapeutic resistance. We aimed to identify its clinical significance as a biomarker for the diagnosis and prediction of breast cancer.

**Methods:**

Western blot, ELISA and in silico analysis were used to confirm CPT1A levels in breast cancer cell lines, cell culture medium and breast cancer tissues. Four hundred thirty breast cancer patients, 200 patients with benign breast disease, and 400 healthy controls were enrolled and randomly divided into a training set and a test set with a 7:3 ratio. Training set was used to build diagnostic models and 10-fold cross validation was used to demonstrate the performance of the models. Then test set was aimed to validate the effectiveness of the diagnostic models. ELISA was conducted to detect individual serum CPT1A levels. Receiver operating characteristic (ROC) curves were generated, and binary logistic regression analyses were performed to evaluate the effectiveness of CPT1A as a biomarker in breast cancer diagnosis. CPT1A levels between post-operative and pre-operative samples were also compared.

**Results:**

CPT1A was overexpressed in breast cancer tissues, cell lines and cell culture medium. Serum CPT1A levels were higher in breast cancer patients than in controls and were significantly associated with metastasis, TNM stage, histological grading and molecular subtype. CPT1A levels were decreased in post-operative samples compared with paired pre-operative samples. Moreover, CPT1A exhibited a higher efficacy in differentiating breast cancer patients from healthy controls (training set: area under the curve, AUC, 0.892, 95% CI, 0.872–0.920; test set, AUC, 0.904, 95% CI, 0.869–0.939) than did CA15–3, CEA, or CA125.

**Conclusion:**

CPT1A is overexpressed in breast cancer and can be secreted out of breast cancer cell. Serum CPT1A is positively associated with breast cancer progression and could serve as an indicator for disease monitoring. Serum CPT1A displayed a remarkably high diagnostic efficiency for breast cancer and could be a novel biomarker for the diagnosis of breast cancer.

**Supplementary Information:**

The online version contains supplementary material available at 10.1186/s12885-021-08134-7.

## Background

Breast cancer is the leading cause of cancer-related death among women. In 2018, 2.1 million new breast cancer cases and 0.6 million cancer-related deaths were estimated worldwide, accounting for 24.4% of total new cancer cases and 15% of total cancer deaths in women [1]. In China, there were an estimated 268,600 new female breast cancer cases and 69,500 deaths in 2015 [2]. In the last two decades, the incidence of breast cancer in China has increased twice as fast as the global rate, particularly in urban areas, because of the prevalence of obesity, physical inactivity, and changes in reproductive pattern [3]. Although early-stage breast cancer is associated with a favorable prognosis, patients diagnosed at an advanced stage suffer from metastasis, therapeutic relapse and poor outcomes [4–6]. In view of the huge burden of breast cancer, there is an increasing demand for improved screening, diagnosis, and management of this disease.

The most widely used method for breast cancer screening is mammography [7, 8]. Although this method was reported to reduce cancer-specific mortality, it has the potential harm of overdiagnosis, which can lead to unnecessary treatment and pain [9, 10]. Further diagnosis of primary breast cancer is based on histopathological examination of tumor tissue [11, 12]. However, these invasive methods are painful, time-consuming, and sometimes tumoral heterogeneity leads to inaccurate diagnoses. Thus, it is important to develop novel, non-invasive and rapid diagnostic methods for breast cancer.

Tumor-specific proteins produced by cancer cells can be identified in the blood of cancer patients and used for cancer diagnosis [13]. At present, carcinoembryonic antigen (CEA) and cancer antigen 15–3 (CA15–3) are the most widely used serum tumor markers for the detection and assessment of treatment responses in breast cancer [14]. Cancer antigen 125 (CA125), which is primarily used for detection of ovarian cancer, has also been suggested as a tumor marker for breast cancer [15]. However, the sensitivity of these biomarkers is limited in the early stages of breast cancer [16].

Carnitine palmitoyl transferase 1A (CPT1A) controls the rate-limiting step of fatty acid oxidation (FAO) which is increasing recognized as crucial metabolic signature of cancer [17–23]. Recently CPT1A is emerged as a crucial regulator for breast cancer [24–29]. CPT1A is upregulated in MYC-overexpressing triple-negative breast cancer (TNBC), radiation-resistant breast cancer cells and radiation-derived breast cancer stem cells [26, 27]. Inhibition of CPT1A activates cell apoptosis and suppresses cell invasion in breast cancer [26, 27, 29]. Overall, these studies indicated that CPT1A plays an important role in breast cancer progression and could be a promising target in breast cancer.

Our previous study found that CPT1A promotes radiation-resistance in nasopharyngeal carcinoma, and gene ontology enrichment analysis showed that exocytosis is predicted as the most associated biological process for CPT1A-binding proteins [22]. Moreover, CPT1A was identified in extracellular vesicles (EVs) derived from breast, ovary, kidney cancer and leukemia cell lines by using proteomic profiling in two studies [30, 31]. These studies indicated that, although CPT1A is known to locate on mitochondrial outer membranes [32], it might also exist in EVs and play a role in EVs-mediated biological activities in cancer.

In this study, we aimed to identify the clinical significance of CPT1A as a biomarker for the diagnosis and prediction of breast cancer. We confirmed the expression level of CPT1A in breast cancer tissues, cell lines and culture medium. Then, we measured the serum levels of CPT1A in a large-scale study and evaluate its clinical significance and diagnostic efficiency for breast cancer.

## Methods

### Patients

In this large-scale study, a total of 560 breast cancer patients, 280 patients with benign breast disease and 600 healthy women were included from the Central Hospital of Wuhan between March 2017 and January 2019. Blood samples of patients were collected 1 day before surgery or breast puncture biopsy. The diagnosis of breast cancer and benign breast disease was confirmed by histopathology according to the guidelines of the National Comprehensive Cancer Network and the guidelines of the Chinese Society of Clinical Oncology.

Breast cancer patients were enrolled according to the following criteria: female patients were newly diagnosed with breast cancer and were not subjected to any other malignant disease, severe injury, or anti-neoplastic therapy before the diagnosis. We excluded 130 breast cancer patients from the study: 39 patients did not have certain histopathological diagnosis, 25 patients had breast tumor metastasized from other organs, 35 patients had received chemotherapy, 11 patients were suffered from breast cancer recurrence, and 20 patients were lost to follow-up. Finally, 430 patients newly diagnosed with breast cancer were enrolled.

Controls were cancer-free subjects, consisted of healthy controls and individuals with benign breast disease. Healthy individuals without exposure to potentially harmful chemicals and malignant disease were verified to be healthy after routine diagnostic tests, including chest X-rays, liver and kidney function tests, viral index analysis and tumor marker analysis, at the time of blood collection. Eighty-five individuals were excluded for dyslipidemia, 87 individuals were excluded for liver dysfunction and 28 individuals were excluded for coagulation disorders. Thus, 400 healthy individuals were enrolled as healthy control. Patients with benign breast disease who were not subjected to malignant disease, severe injury and acute inflammation were enrolled. Twenty-four patients were excluded because of a history of cancer, 18 patients were excluded for lactational mastitis, 22 patients were excluded for breast prosthesis-caused inflammation and 16 patients were excluded for loss to follow-up. As a result, 200 patients with benign breast disease were included.

The finally included participants were randomly divided into a training and test sets with a 7:3 ratio. The training set was used to develop the diagnostic models and the test set was aimed to validate the effectiveness of the diagnostic models. In training set, 126 paired breast cancer serum samples were collected before surgery and 1 week after surgery, to assess tumor-monitoring value. In test set, 44 paired pre-and post-treatment breast cancer serum samples were collected. Clinicopathologic information about patients, including age, pathology, tumor node metastasis (TNM) stage, and molecular subtype, was obtained from hospital pathologic records (Table 1).

**Table 1:** The characteristics of the patient population

Clinical parameters	Training set			Test set		
	CPT1A level			CPT1A level		
	High	Low	*p* Value	High	Low	*p* Value
Age						
≤ 60	85 (28.2)	95 (31.6)	0.269	43 (33.3)	48 (37.2)	0.407
> 60	65 (21.6)	56 (18.6)		21 (16.3)	17 (13.2)	
Menopausal status, n (%)						
Premenopausal	76 (25.2)	85 (28.2)	0.328	35 (27.1)	40 (31.0)	0.430
postmenopausal	74 (24.6)	66 (21.9)		29 (22.5)	25 (19.4)	
Tumor size, n (%)						
Tis	5 (3.9)	20 (15.5)	0.000	4 (3.1)	8 (7.0)	0.033
T1	44 (34.1)	52 (40.3)		16 (12.4)	30 (23.3)	
T2	84 (65.1)	76 (58.9)		35 (27.1)	24 (18.6)	
T3	13 (10.1)	3 (2.3)		7 (5.4)	3 (2.3)	
T4	4 (3.1)	0 (0.0)		2 (1.6)	0 (0.0)	
Lymph node status, n (%)						
N0	75 (24.9)	108 (35.9)	0.001	33 (25.6)	49 (38.0)	0.004
N1	39 (13.0)	24 (8.0)		12 (9.3)	11 (8.5)	
N2	17 (5.6)	13 (4.3)		7 (5.4)	4 (3.1)	
N3	19 (6.3)	6 (2.0)		12 (9.3)	1 (0.8)	
TNM stage, n (%)						
0	5 (1.7)	20 (6.6)	0.000	4 (3.1)	8 (6.2)	0.000
I	27 (9.0)	41 (13.6)		6 (4.7)	25 (19.4)	
IIA	48 (15.9)	53 (17.6)		24 (18.6)	15 (11.6)	
IIB	25 (8.3)	19 (6.3)		7 (5.4)	10 (7.8)	
III	39 (13.0)	15 (5.0)		19 (14.7)	6 (4.7)	
IV	6 (2.0)	3 (1.0)		4 (3.1)	1 (0.8)	
Histological grading, n (%)						
G1	9 (3.0)	22 (7.3)	0.045	2 (1.6)	15 (11.6)	0.000
G2	82 (27.2)	81 (26.9)		25 (19.4)	33 (25.6)	
G3	56 (18.6)	47 (15.6)		35 (27.1)	16 (12.4)	
Unkown	3 (1.0)	1 (0.3)		2 (1.6)	1 (0.8)	
ER, n (%)						
Negative	64 (21.3)	61 (20.3)	0.690	13 (10.1)	22 (17.1)	0.063
Positive	86 (28.6)	90 (29.9)		51 (39.5)	42 (33.3)	
PR, n (%)						
≤ 20	71 (23.6)	79 (26.2)	0.387	28 (21.7)	21 (16.3)	0.181
> 20	79 (26.2)	72 (23.9)		36 (27.9)	44 (34.1)	
HER2, n (%)						
Negative	108 (35.9)	85 (28.2)	0.004	47 (36.4)	36 (27.9)	0.032
Positive	42 (14.0)	66 (21.9)		17 (13.2)	29 (22.5)	
Ki67, n (%)						
< 15%	20 (6.6)	33 (11.0)	0.052	14 (10.9)	20 (15.5)	0.252
≥ 15%	130 (43.2)	118 (39.2)		50 (38.8)	45 (34.9)	
Molecular subtype, n (%)						
Luminal A	11 (3.7)	18 (6.0)	0.000	7 (5.4)	15 (11.6)	0.033
Luminal B/HER2-	59 (19.6)	52 (17.3)		23 (17.8)	33 (25.6)	
Luminal B/HER2+	16 (5.3)	21 (7.0)		12 (9.3)	9 (7.0)	
HER2+	23 (7.6)	44 (14.6)		9 (7.0)	4 (3.1)	
TNBC	41 (13.6)	16 (5.3)		13 (10.1)	4 (3.1)	

Pearson’s chi-square test

### Sample collection

Blood samples were collected in the morning before breakfast with informed consent from patients. Four millilitre peripheral blood samples were collected in separation gel/coagulation-promoting vacuum tubes. The samples were centrifuged at 2, 000 x g for 15 min at 4 °C within 1 h after collection. The supernatants (sera) were then transferred into new tubes and stored at − 80 °C until testing.

### Cell lines

The human immortalized mammary epithelial cell line HBL-100 (Cat. # GNHu10), human breast cancer cell lines SK-BR-3 (Cat. # TCHu225), T-47D (Cat. # TCHu87), MCF7 (Cat. # TCHu74), MDA-MB-453 (Cat. # SCSP-5044), MDA-MB-468 (Cat. # TCHu136), MDA-MB-231 (Cat. # TCHu227), Hs578T (Cat. # TCHu127) and BT549 (Cat. # TCHu93) were purchased from the Cell Bank of the Chinese Academy of Science (Shanghai, China) in 2016. Cells were incubated in a humidified incubator with 5% CO_2_ at 37 °C. HBL-100, SK-BR-3 and T-47D cells were cultured in Dulbecco’s Modified Eagle’s medium (DMEM, Hyclone, USA) containing 10% fetal bovine serum (FBS, Gibco, USA). MCF7 cells were cultured in Eagle’s minimum essential medium (EMEM, Hyclone, USA) containing 10% FBS and 0.01 mg/mL bovine insulin. MDA-MB-453, MDA-MB-468 and MDA-MB-231 cells were cultured in Leibovitz’s L-15 medium containing 10% FBS. HCC1806 cells were cultured in PRMI-1640 medium containing 10% FBS. Hs578T cells were cultured in DMEM medium with 0.01 mg/ml insulin and 10% FBS. BT549 cells were cultured in PRMI-1640 medium with 0.0231 units/mL insulin and 10%FBS. Cells were routinely authenticated every year by using the following methods: cellular DNA was purified with QIAamp DNA mini kit (QIAGEN, Cat. # G51306) and used for PCR amplification with STR Multi-amplification kit (Goldeneye DNA ID System 20A, Peoplespot), then the PCR products were assayed with 3100xI DNA Analyzer (Applied Biosystems). Cells were routinely tested for mycoplasma contamination using the PCR Mycoplasma Detection Kit (ABM, Cat. # G238). Cells were grown in T25 culture flasks and passaged using 0.25% Trypsin/EDTA. Protein and cell culture medium were collected within 3 passages in 2 weeks.

### Western blot analysis

Cell pellets were harvested and disrupted in IP lysis buffer (Thermo Scientific, MA, USA). Protein concentrations were measured using a BCA assay kit (Thermo Scientific, MA, USA). Then proteins were separated by SDS-PAGE and transferred onto a 0.45-μm PVDF membrane at 4 °C for 1 h (Millipore, USA). Then 5% fatty acids-free milk was used to incubate with PVDF membranes at room temperature for 2 h. After that, CPT1A primary antibody (ab102679, Abcam, MA, USA) was used as 1:1000 and β-Actin primary antibody (A2066, Sigma-Aldrich, Darmstadt, Germany) was used as 1:3000 to incubate with PVDF membranes at 4 °C overnight. Wash the membranes with PBST buffer for 3 times and 10 min for each time. Then peroxidase-conjugated secondary antibody (7076, Cell Signaling Technology, MA, USA) were used as 1:1000 to incubate with the membranes at room temperature for 1 h. Wash the membranes with PBST buffer for 3 times and 10 min for each time. Visualization and grayscale analysis were performed by using the ChemiDoc XRS system and Image Lab software (Bio-Rad, CA, USA).

### Elisa

CPT1A levels of serum and cell culture medium were detected by using a commercially available ELISA kit (SEH368Hu96 Test, Cloud-Clone Corp, China) according to the manufacturer’s protocol. This kit is a sandwich enzyme immunoassay for quantitative measurement of CPT1A. serum samples were diluted in Standard Diluent as 1:5, 1:10, and 1:20 in a preliminary experiment. Then we chose the optimal sample dilution as 1:10, to guarantee the values were within the range of the standard curve. Standards, diluted serum samples and cell culture medium were run in triplicate. Blank, diluted standard series and samples in a 100 μL total volume were added to the wells of an ELISA analysis plate pre-coated with an antibody specific to CPT1A. The plate was covered with a sealer and incubated for 1 h at 37 °C. Then the liquid was removed, and 100 μL of Reagent A, containing a biotin-conjugated antibody specific to CPT1A, was added to each well. The plate was incubated for another 1 h at 37 °C and washed with Wash Buffer three times. Then 100 μL of Reagent B, containing avidin-conjugated horseradish peroxidase (HRP) was added, and the plate was incubated for 30 min at 37 °C. After five washes with Wash Buffer, a 3, 3, 5, 5-tetramethylbenzidine (TMB) substrate which reacted with the HRP enzyme was added to the plates resulting in color development. Then the plate was incubated for 15 min at 37 °C, and the reaction was terminated by addition of a sulfuric acid solution. The optical density (OD) was immediately measured at a wavelength of 450 nm in a plate reader (EnSpire 2300, PerkinElmer, USA). The concentration of CPT1A was determined by curve-fitting to an OD standard curve.

### Logistic regression models

The standard logistic regression formula is:

$$ {\displaystyle \begin{array}{l}\mathrm{Logit}\ \left(\mathrm{P}\right)=\upbeta 0+\upbeta 1\mathrm{X}1+\upbeta 2\mathrm{X}2+\dots \dots +\upbeta \mathrm{nXn},\\ {}\mathrm{Logit}\ \left(\mathrm{P}\right)=\ln \left[\mathrm{p}/\left(1-\mathrm{p}\right)\right]\end{array}} $$

“p” is the estimated probability of breast cancer patients, “β0” is a constant, “β” is the influence coefficient, “n” is the number of influence factors [33, 34].

Formula for predicting breast cancer was developed based on the data of breast cancer patients and controls in the training set. β0 and βn were obtained by binary logistic regression. The estimated probability of breast cancer patients and controls in the test set were calculated using the formula. Then ROC curve analysis and binary logistic regression analysis were conducted based on the estimated probability in the test set to evaluate the effectiveness of the models.

### Analysis of tumor markers and lipids

Serum CA15–3, CEA, and CA125 levels were detected by using an automatic electrochemistry luminescence immunoassay system (Abbott, I2000–2, USA). Serum lipids, including triglyceride (TG), total cholesterol (TC), high-density lipoprotein-cholesterol (HDL-C), low-density lipoprotein-cholesterol (LDL-C), and non-esterified fatty acids (NEFA), were tested using an automatic biochemical analysis system (Olympus, AU5421, Japan). The reference changes of each tumor biomarker and lipid mentioned above are listed in Table S3.

### Survival curve analysis

The Kaplan–Meier method was used to estimate overall survival by log-rank test according to data from the Cancer Genome Atlas (TCGA). The Kaplan–Meier curves were drawn by using the GraphPad Prism 5 software (GraphPad Software, La Jolla, CA, USA).

### Ten-fold cross validation

To avoid over-fitting, a 10-fold cross validation was performed. For 10-fold cross validation, the samples were randomly divided into two parts: one for training and anther for testing. This process was repeated for 10 times. The program was run by using Pathon 3.8. The code is provided on the public code-sharing website scikit-learn (https://scikit-learn.org/stable/index.html). The cross-validated ROC curves were indicated in Fig. S5.

### Statistical analysis

The Mann–Whitney U test was performed to compare the differences between groups using continuous-variable and nonparametric analyses in GraphPad Prism Windows (version 5). Association between CPT1A levels and clinicopathological characteristics were estimated by a Chi-square test. The differences of CPT1A level between pre-surgery and post-surgery serum samples were analyzed by Mann–Whitney U test in GraphPad Prism 5 software. ROC curves were generated to access the sensitivity, specificity and AUCs with a 95% CI of CPT1A or tumor markers in distinguishing breast cancer patients from controls. To evaluate the diagnostic efficiency of CPT1A alone or CPT1A in combination with CA15–3, CEA, and CA125, formulas were obtained in training set by binary logistic regression analysis. The regression formulas are provided in the Table S2. Then ROC curve analysis and binary logistic regression analysis were conducted in the test set by using the formulas to validate the effectiveness of the models. ROC curve analysis and logistic regression analysis were performed using SPSS windows (version 19.0). All *p*-values are two-sided, and *p* < 0.05 is considered to be statistically significant.

## Results

### CPT1A levels in breast cancer tissues, cell lines and culture medium

**Fig. 1:**
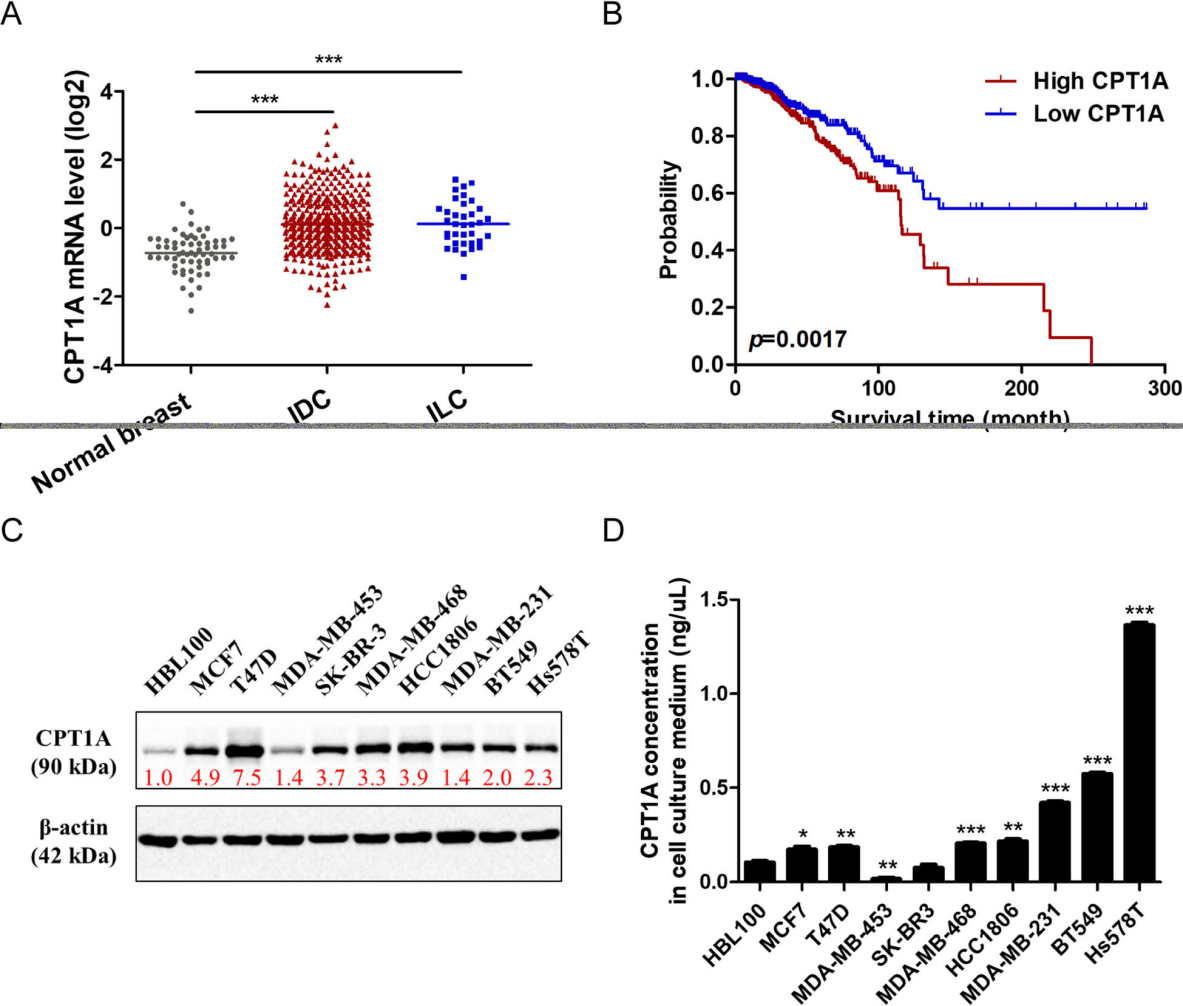
CPT1A levels in breast cancer tissues, cell lines and culture medium. **a** CPT1A mRNA levels in normal breast tissues (*n* = 61), invasive ductal breast cancer (IDC) tissues (*n* = 389) and invasive lobular breast cancer (ILC) tissues (*n* = 36) according to data from the Cancer Genome Atlas (TCGA) database. **b** Overall survival rates of breast cancer patients with low (*n* = 481) or high (*n* = 481) expression levels of CPT1A were estimated with the Kaplan–Meier method by log-rank test according to data from TCGA database. The Kaplan–Meier curves were drawn by using the GraphPad Prism 5 software. **c** CPT1A protein levels in immortalized breast epithelial cell line and breast cancer cell lines. β-Actin was used as a control to confirm equal loading of protein. Greyscale values of each protein bands were calculated. The relative fold change of CPT1A band relative to β-Actin band were indicated below CPT1A bands. Corresponding uncropped full-length blots are presented in Supplementary Fig. 1. The full-length blots were cropped between 55 KDa and 72 KDa to separate CPT1A and β-actin protein. **d** CPT1A protein levels in cell culture medium of indicated cell lines above were detected by an ELISA assay after 72 h incubation. *p < 0.05, **p < 0.001, ***p < 0.0001

Our previous study found that CPT1A binds to several vesicular trafficking proteins, and CPT1A was indeed identified in exosomes of breast cancer cell line in another study [22, 30]. Then we moved to investigate CPT1A levels in breast cancer tissues, cell lines and cell culture medium. We found that CPT1A is overexpressed in breast cancer tissues compared with normal breast tissues. High level of CPT1A leads to a poor outcome of breast cancer patients according to the TCGA database (Fig. 1a, b). CPT1A expression levels are also elevated in breast cancer cell lines compared with immortalized mammary epithelial cells (Fig. 1c, Fig. S1). Then we analyzed the protein level of CPT1A in cell culture medium of a panel of breast cancer cell lines. We found that CPT1A levels in cell culture medium are increased in most breast cancer cell lines, especially in TNBC cell lines, compared with immortalized mammary epithelial cells (Fig. 1d). These results show that CPT1A is overexpressed in breast cancer and could be secreted extracellularly by breast cancer cells, which might be possible to act as a biomarker for breast cancer.

### CPT1A serum levels in patients with breast cancer, benign breast disease and healthy controls

The enrollment and inclusion of participants is shown in Methods and Fig. S2. All the participants were randomly divided into a training set and a test set with a 7:3 ratio. The training set includes 301 breast cancer patients, 280 healthy controls and 140 patients with benign breast disease. The test set includes 129 breast cancer patients, 120 healthy controls and 60 patients with benign breast disease. The characteristics of breast cancer patients are shown in Table 1. The training and test set populations were comparable with respect to most of the clinical parameters.

**Fig. 2:**
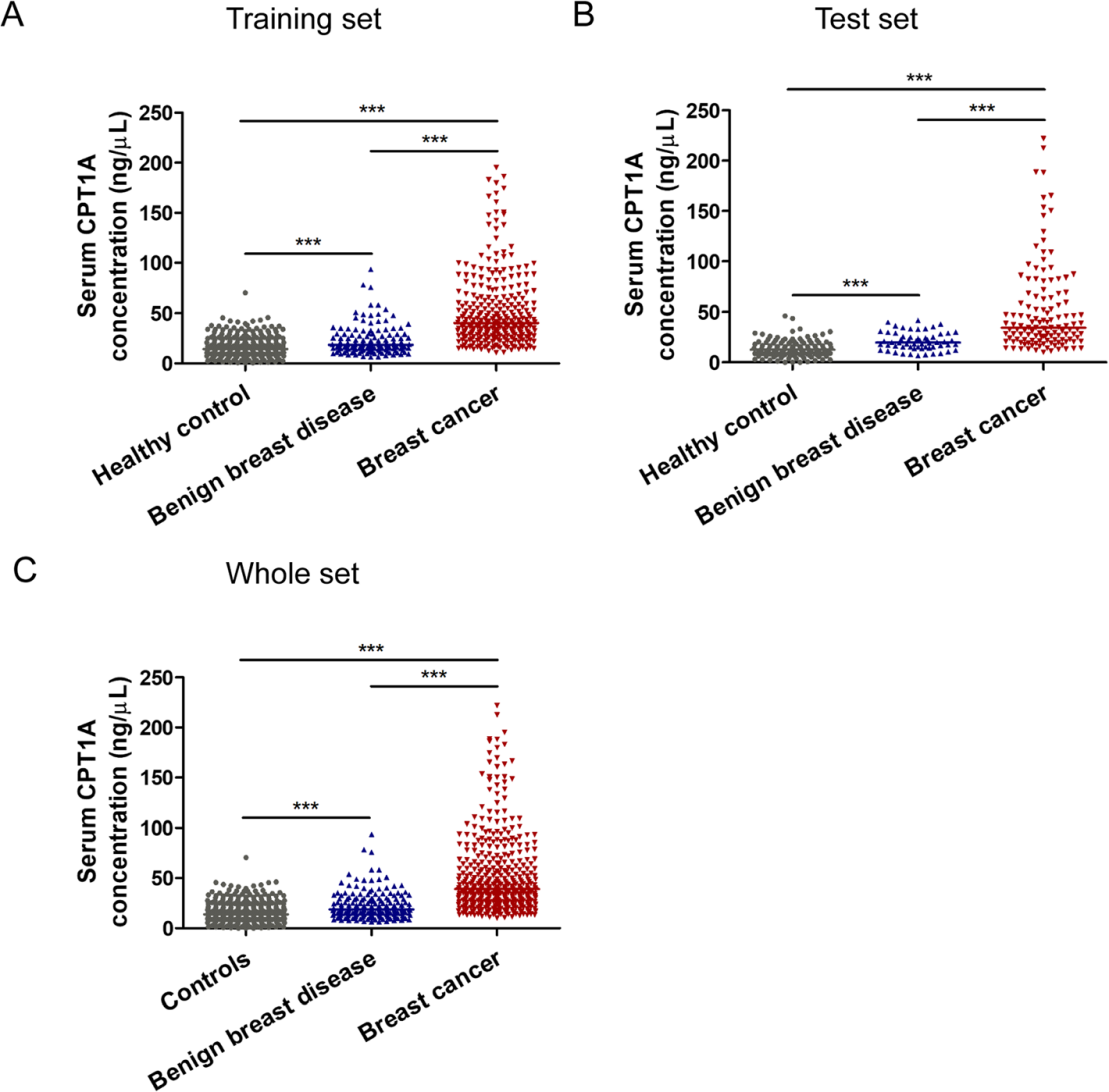
The serum CPT1A levels from healthy controls, patients with benign breast diseases and breast cancer. **a** Serum CPT1A levels in the training set. **b** Serum CPT1A levels in the test set. **c** Serum CPT1A levels in the whole set. The horizontal lines indicated median values. Statistical significance was determined by the Mann–Whitney U test. **p < 0.001, ***p < 0.0001

We analyzed the serum CPT1A levels (median ± IQR; IQR, interquartile range) in each participant using an ELISA assay. In the training set, the median serum CPT1A level in breast cancer patients was 40.22 ± 35.12 ng/mL, which was significantly higher than that of patients with benign breast disease (18.44 ± 14.61 ng/mL; *p* < 0.0001) or healthy controls (14.37 ± 14.56 ng/mL; Fig. 2a, *p* < 0.0001). In the test set, the median CPT1A level in breast cancer patients was 34.56 ± 42.31 ng/mL, which was notably higher than that of patients with benign breast disease (19.64 ± 12.33 ng/mL; *p* < 0.0001), or healthy controls (12.53 ± 11.43 ng/mL; Fig. 2b, *p* < 0.0001). In the whole set, the median CPT1A levels in serum from breast cancer patients was 38.99 ± 36.63 ng/mL, which was significantly higher than the levels observed in patients with benign breast disease (18.63 ± 13.91 ng/mL; *p* < 0.0001) or healthy controls (13.79 ± 11.97 ng/mL; Fig. 2c, *p* < 0.0001).

### The relationship between serum CPT1A level and the clinicopathological characteristics of breast cancer patients

To evaluate the clinical significance of serum CPT1A, correlation between serum CPT1A level and clinical parameters 0f breast cancer patients were estimated by a Pearson’s Chi-square test (Table 1). The threshold value used to classify breast cancer patients into the high and low groups is the median value of CPT1A level in each set. We found that CPT1A level is significantly associated with lymph node status, tumor size, TNM stage, histological grading, human epidermal growth factor receptor 2 (HER2) status and molecular subtype in both the training and test set (Table 1).

**Fig. 3:**
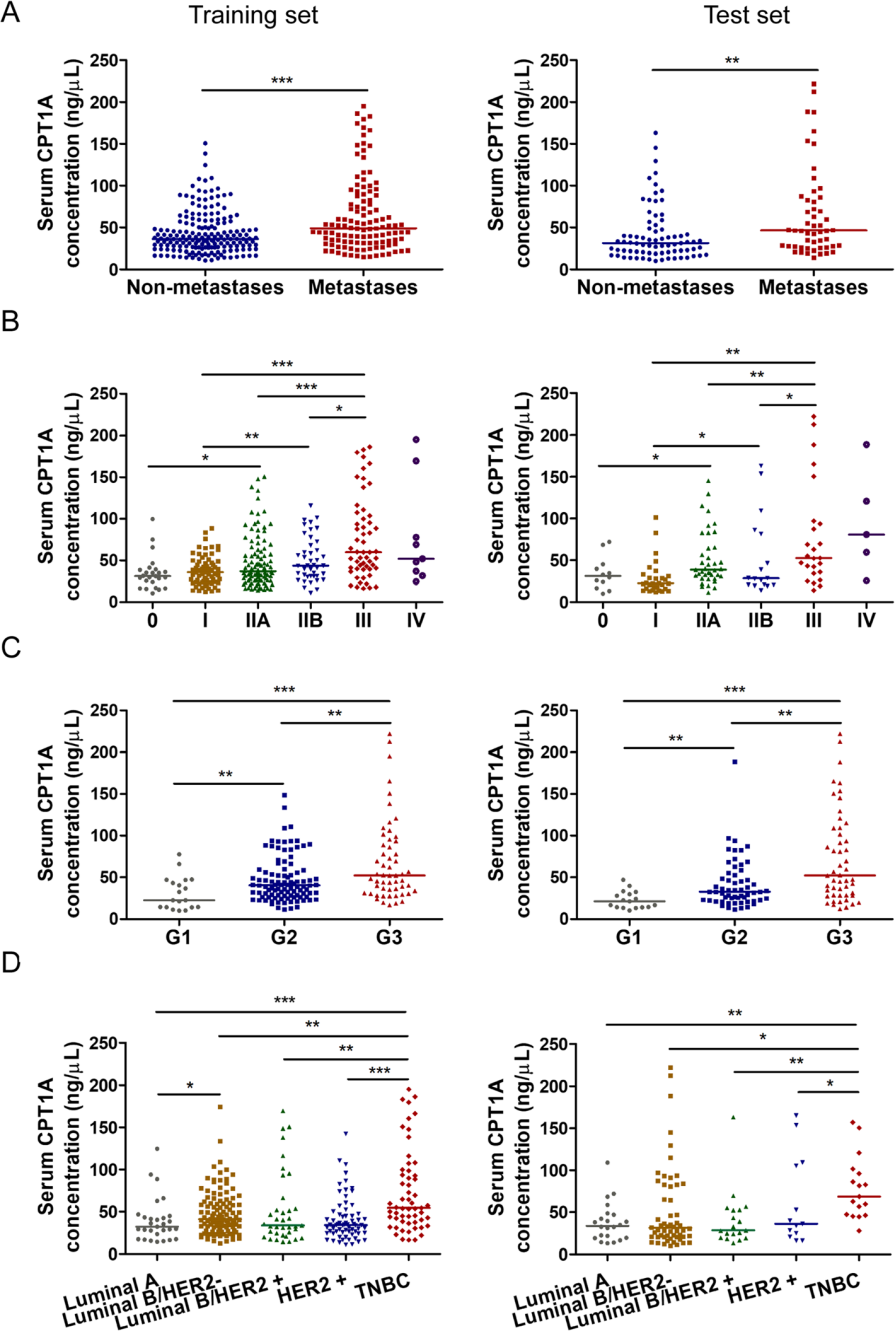
Serum CPT1A level in different clinicopathological classifications of breast cancer. **a** Serum CPT1A levels from non-metastatic and metastatic breast cancer patients. **b** Serum CPT1A levels from breast cancer patients with different TNM stage. **c** Serum CPT1A levels from breast cancer patients with different histological grade. **d** Serum CPT1A levels from breast cancer patients of different molecular subtype. Statistical significance was were determined by the Mann–Whitney U test. *p < 0.05, **p < 0.001, ***p < 0.0001

The results also showed that the median value of CPT1A level in patients with lymph node or distant metastasis (training set: 48.77 ± 53.41 ng/mL; test set: 46.26 ± 57.74 ng/mL) was significantly higher than in patients without metastasis (training set: 36.23 ± 28.87 ng/mL, *p* < 0.0001; test set, 31.15 ± 27.80 ng/mL, *p* < 0.001; Fig. 3a). Relative to in situ cancer cases (training set: 31.17 ± 18.35 ng/mL; test set: 31.20 ± 25.37 ng/mL), invasive ductal carcinoma cases exhibited higher CPT1A levels (training set: 41.21 ± 36.66 ng/mL, *p* < 0.001; test set: 37.90 ± 42.91 ng/mL, *p* < 0.001; Fig. S3). Patients with advanced TNM stage III exhibited higher CPT1A serum levels (training set: 59.89 ± 63.82 ng/mL; test set: 52.68 ± 59.77 ng/mL), compared with patients with stage I (training set: 36.12 ± 24.34 ng/mL, *p* < 0.0001; test set: 22.46 ± 15.23 ng/mL, *p* < 0.001), II A (training set: 36.98 ± 35.70 ng/mL, *p* < 0.0001; test set: 38.61 ± 34.50 ng/mL, *p* < 0.001), or II B (training set: 43.73 ± 38.10 ng/mL, *p* < 0.05; test set: 28.41 ± 63.19 ng/mL, *p* < 0.05; Fig. 3b). We also found that serum CPT1A levels were remarkably higher in histological grade 3 (G3) cases (training set: 45.86 ± 51.96 ng/mL; test set: 52.16 ± 80.69 ng/mL) than in grade 1 (G1) cases (training set: 32.12 ± 29.60 ng/mL, *p* < 0.0001; test set: 21.17 ± 17.09 ng/mL, *p* < 0.0001) and grade 2 (G2) cases (training set: 40.22 ± 28.55 ng/mL, *p* < 0.001; test set: 32.69 ± 30.70 ng/mL, *p* < 0.001; Fig. 3c). Moreover, significant elevation of CPT1A levels was observed in triple-negative breast cancer patients (training set: 54.55 ± 58.06 ng/mL; test set: 59.56 ± 48.23 ng/mL) relative to luminal A (training set: 32.12 ± 23.64 ng/mL, *p* < 0.0001; test set: 33.66 ± 26.12 ng/mL, *p* < 0.001), luminal B/HER2^−^ (training set: 41.09 ± 31.59 ng/mL, *p* < 0.001; test set: 31.34 ± 61.74 ng/mL, *p* < 0.05), luminal B/HER2^+^ (training set: 33.73 ± 36.63 ng/mL, *p* < 0.001; test set: 28.43 ± 33.27 ng/mL, *p* < 0.001), and HER2^+^ patients (training set: 34.15 ± 22.81 ng/mL, *p* < 0.0001; test set: 39.94 ± 100.39 ng/mL, *p* < 0.05; Fig. 3d).

In addition, we did not find any correlation between serum CPT1A level and age, menopausal status, or ki-67 level in either training or test sets (Table 1). As CPT1A is a key regulator in lipid metabolism, we analyzed the correlation between serum levels of CPT1A and lipids (TG, TC, HDL-C, LDL-C, and NEFA) in the diagnosis of breast cancer. Spearman correlation analysis indicated no correlation between CPT1A and TG, TC, HDL-C, LDL-C, or NEFA level respectively (Fig. S4). Moreover, univariate logistic regression analysis also revealed that CPT1A was an effective diagnostic factor independently of the lipids in differentiating breast cancer patients from healthy controls (Table S1).

### Construction and validation of diagnostic models for breast cancer

**Fig. 4:**
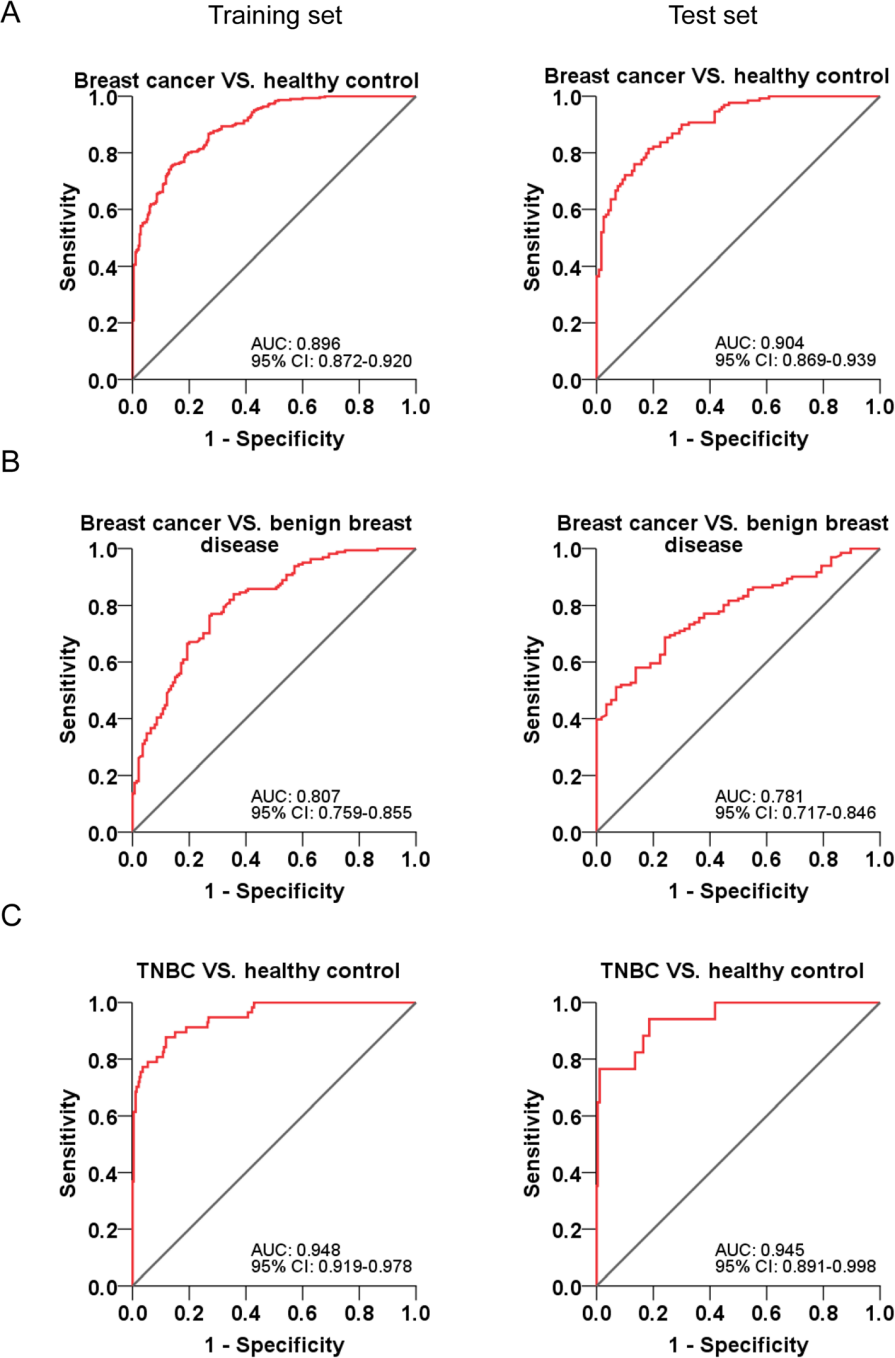
ROC curve analyses using CPT1A to differentiate breast cancer from benign breast disease or healthy controls. **a** ROC curves of CPT1A for breast cancer patients versus healthy controls in the training set (left) and test set (right), respectively. **b** ROC curves of CPT1A for breast cancer patients versus benign breast cancer patients in the training set (left) and test set (right), respectively. **c** ROC curves of CPT1A for TNBC patients versus healthy controls in the training set (left) and test set (right), respectively

ROC curves based on the ELISA results in the training set and test set were plotted to determine the diagnostic efficiency of serum CPT1A in breast cancer (Fig. 4, Table 2). In the training set, the AUC of CPT1A in differentiating breast cancer patients from healthy controls was 0.892 (Fig. 4a, left; 95% CI, 0.872–0.920), and the optimum CPT1A cutoff value was 27.57 ng/mL for breast cancer diagnosis (training set: sensitivity, 75.4%; specificity, 86.1%). We also found that CPT1A exhibited good efficacy in differentiating patients with breast cancer from patients with benign breast disease (Fig. 4b, left; AUC, 0.807; 95% CI, 0.759–0.855). Moreover, CPT1A displayed an extremely high discriminatory capacity for differentiating TNBC patients from healthy controls (Fig. 4c, left; AUC, 0.948; 95% CI, 0.919–0.978).

**Table 2:** The diagnostic efficiency of models in differentiating breast cancer and controls

	Training set							Test set						
	AUC (95% CI)	SN (%)	SP (%)	PPV (%)	NPV (%)	+LR	-LR	AUC (95% CI)	SN (%)	SP (%)	PPV (%)	NPV (%)	+LR	-LR
Breast cancer VS. Controls														
CPT1A^a^	0.892 (0.872–0.920)	75.4	86.1	81.8	77.1	5.42	0.29	0.904 (0.869–0.939)	81.4	81.7	84.2	76.0	4.45	0.23
CA153	0.553 (0.506–0.599)	52.8	61.4	56.1	56.8	1.37	0.77	0.548 (0.475–0.620)	62.8	55.0	41.7	68.2	1.40	0.68
CEA	0.608 (0.562–0.654)	69.1	49.3	62.5	51.2	1.36	0.63	0.560 (0.488–0.631)	55.0	62.5	50.0	63.6	1.47	0.72
CA125	0.524 (0.477–0.571)	65.1	42.5	32.1	73.4	1.13	0.82	0.547 (0.475–0.618)	39.5	73.3	45.0	56.6	1.48	0.83
CA153 + CEA + CA125	0.605 (0.559–0.651)	88.7	27.9	60.4	48.2	1.23	0.41	0.581 (0.510–0.652)	82.9	35.0	54.2	58.9	1.28	0.49
CPT1A + CA153 + CEA + CA125	0.904 (0.881–0.927)	76.4	86.1	83.9	77.1	5.50	0.27	0.902 (0.865–0.938)	76.0	90.8	88.3	77.5	8.26	0.26
Breast cancer VS. Benign breast disease														
CPT1A	0.807 (0.759–0.855)	74.6	72.9	75.7	68.3	2.75	0.35	0.781 (0.717–0.846)	68.7	75.9	44.8	85.5	2.85	0.41
TNBC VS. Controls														
CPT1A	0.948 (0.919–0.978)	87.7	88.2	98.2	70.2	7.43	0.14	0.945 (0.891–0.998)	94.1	81.4	99.6	52.9	5.06	0.07

Abbreviations: *+LR* positive likelihood ratio, *−LR* negative likelihood ratio, *PPV* positive predictive value, *NPV* negative predictive value

^a^The diagnostic cut-off value was 26.08 ng/mL

According to the binary logistic regression analysis in the training set, diagnostic models of CPT1A for breast cancer diagnosis were constructed. The formulas were list in Table S2. Then the validation of the logistic regression models was assessed in the test set. As a result, in the test set, the AUC of CPT1A in differentiating breast cancer patients from healthy controls was 0.904 (Fig. 4a, right; 95% CI, 0.869–0.939). Additionally, CPT1A was also effective in differentiating patients with breast cancer from patients with benign breast disease (Fig. 4b, right; AUC, 0.781; 95% CI, 0.717–0.846), and in differentiating TNBC patients from healthy controls (Fig. 4c, right; AUC, 0.945; 95% CI, 0.891–0.998), in the test set. These results show that CPT1A satisfactorily discriminates breast cancer patients from healthy controls or patients with benign breast disease. Moreover, it is exhibited a high discriminatory capacity for differentiating TNBC patients from healthy controls.

**Fig. 5:**
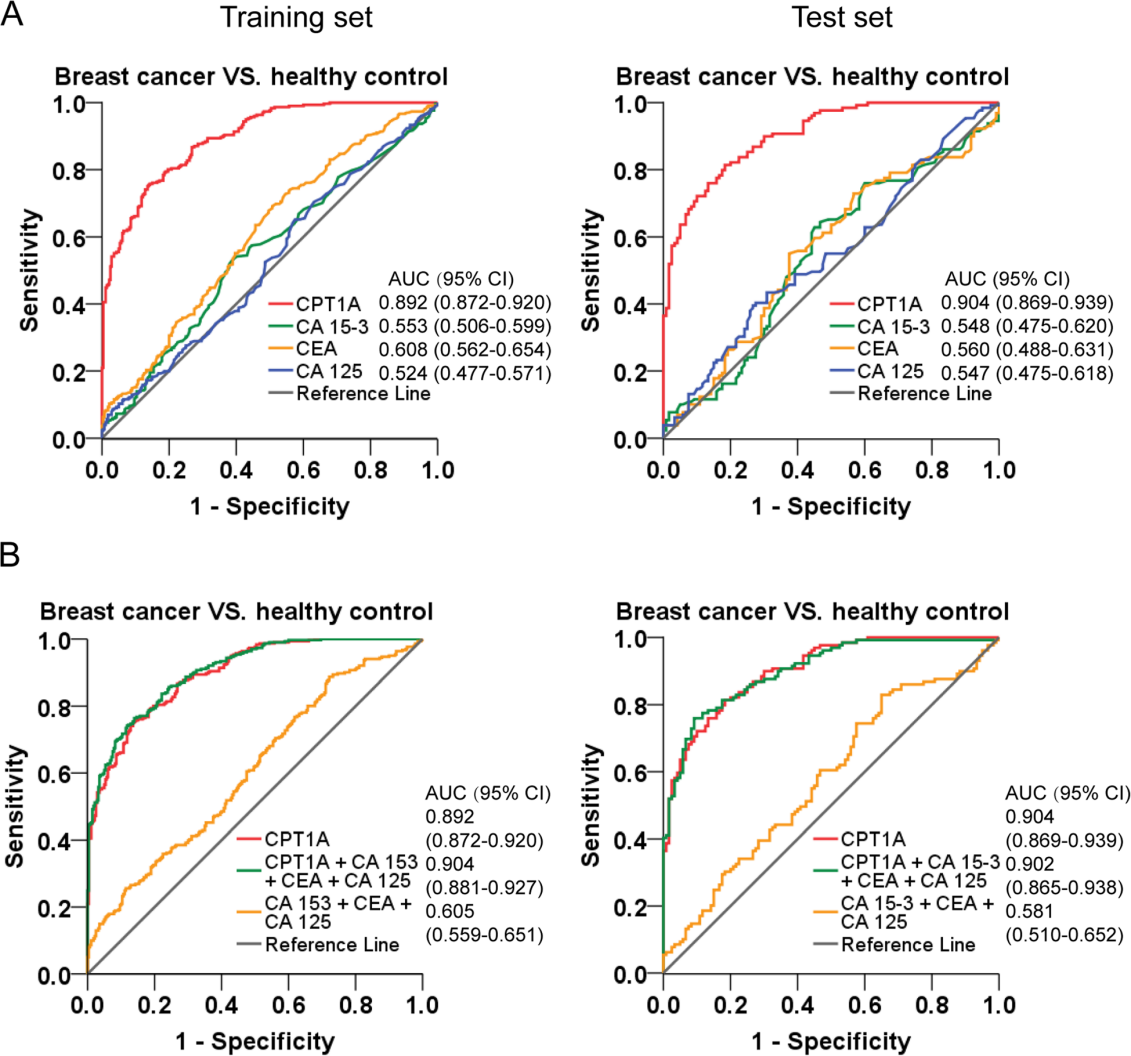
The ROC curve analyses using CPT1A, CA15–3, CEA and CA125 for the differentiation of breast cancer cases and healthy controls. **a** ROC curves of CPT1A, CA15–3, CEA and CA125 for breast cancer cases versus healthy controls in the training set (left) and test set (right), respectively. **b** ROC curves of CPT1A and a model which combined the analysis of CPT1A, CA15–3, CEA and CA125 for breast cancer cases versus healthy controls in the training set (left) and test set (right), respectively

To better understand the potential use of serum CPT1A as a clinical biomarker, we further compared the predictive value and likelihood ratios of CPT1A with three conventional tumor markers, CA15–3, CEA, and CA125, in discrimination of breast cancer patients from healthy controls. Diagnostic models of each marker and combining panels were constructed by binary logistic regression analysis based on the data in the training set. To evaluate the prediction performance of the models, 10-fold cross validation was performed. Among the four biomarkers, CPT1A (training set: AUC, 0.892; 95% CI, 0.872–0.920, Fig. 5a, left; cross-validated AUC, 0.909; 95% CI, 0.874–0.945, Fig. S5A) displayed a significantly higher AUC in differentiating breast cancer patients from healthy controls than did CA15–3 (training set: AUC, 0.553; 95% CI, 0.506–0.599, Fig. 5a, left; cross-validated AUC, 0.557; 95% CI, 0.503–0.611, Fig. S5B), CEA (training set: AUC, 0.608; 95% CI, 0.562–0.654, Fig. 5a, left; cross-validated AUC, 0.605; 95% CI, 0.529–0.680, Fig. S5C), CA 125 (training set: AUC, 0.524; 95% CI, 0.447–0.571, Fig. 5a, left; cross-validated AUC, 0.525; 95% CI, 0.469–0.582, Fig. S5D), and the combination of CA 15–3, CEA and CA 125 (training set: AUC, 0.605; 95% CI, 0.559–0.651, Fig. 5b, left; cross-validated AUC, 0.563; 95% CI, 0.511–0.617, Fig. S5E). However, the combination of CPT1A, CA15–3, CEA, and CA125 (training set: AUC, 0.904; 95% CI, 0.881–0.927, Fig. 5b, left; cross-validated AUC, 0.914; 95% CI, 0.880–0.948; Fig. S5F) did not significantly improve the classification capacity and diagnostic efficacy of breast cancer patients than CPT1A alone.

In addition, the 10-fold cross-validated AUC values showed relatively modest difference from the AUC values we obtained from the established models based on the training set, which confirms that there is no over fitting and the models are reliable. Moreover, the models showed consistent results between training and test set. CPT1A (test set: AUC, 0.904; 95% CI, 0.869–0.939, Fig. 5a, right), and the combination of CPT1A, CA 153, CEA and CA 125 (test set: AUC, 0.902; 95% CI, 0.865–0.938, Fig. 5b, right) displayed a significantly higher AUC in differentiating breast cancer patients from healthy controls than did CA15–3 (test set: AUC, 0.548; 95% CI, 0.475–0.620, Fig. 5a, right), CEA (test set: AUC, 0.560; 95% CI, 0.488–0.618, Fig. 5a, right), CA125 (test set: AUC, 0.547; 95% CI, 0.475–0.618, Fig. 5a, right), and the combination of CPT1A, CA15–3, CEA, and CA125 (test set: AUC, 0.581; 95% CI, 0.510–0.652, Fig. 5b, right).

### Comparison of CPT1A levels in paired pre-and post-operative breast cancer serum samples

**Fig. 6:**
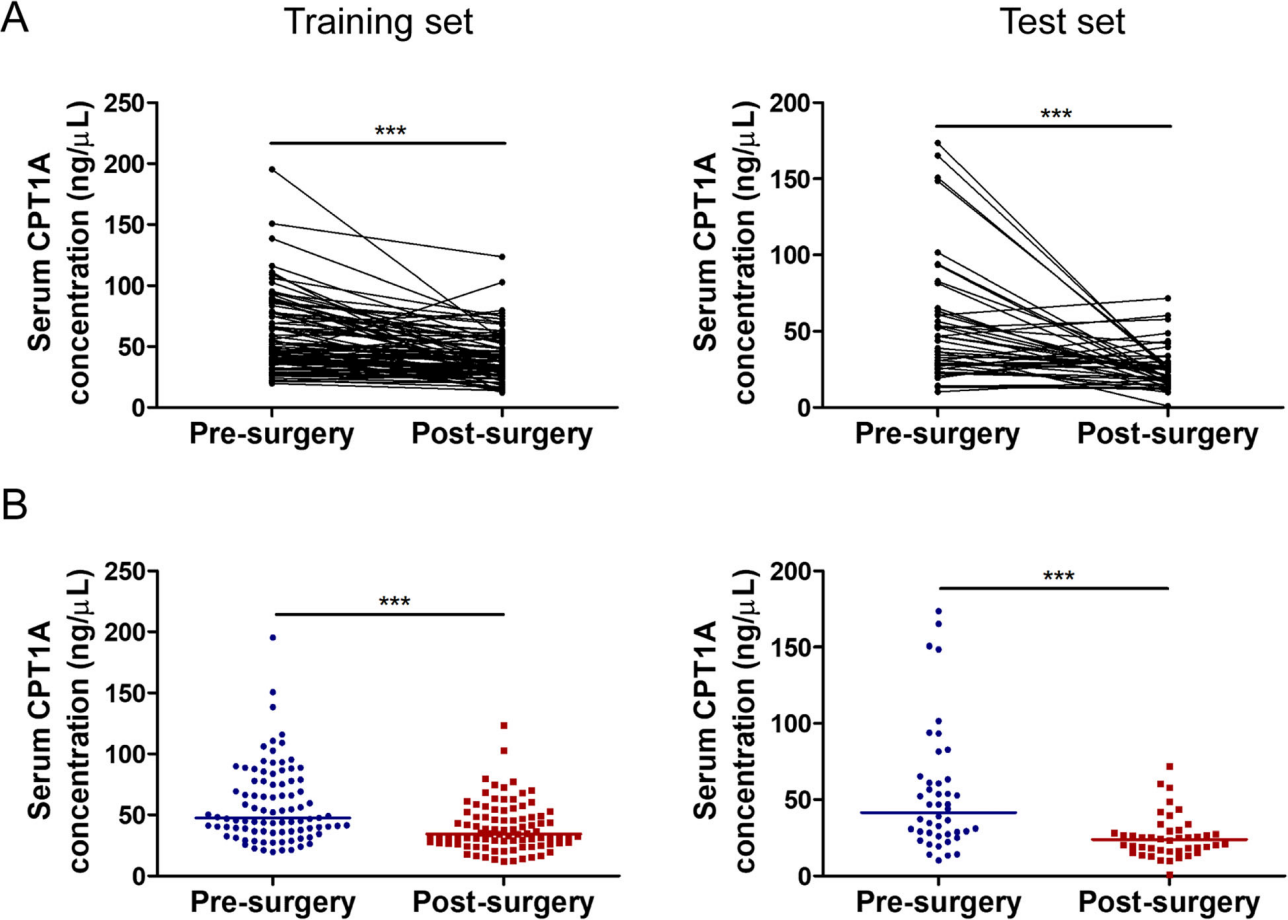
Serum CPT1A levels of pre-surgery and post-surgery breast cancer patients. **a** CPT1A levels in serum from the same breast cancer patient before and after surgery, in the training set (left, *n* = 126) and test set (right, *n* = 44), respectively. **b** Serum CPT1A levels from breast cancer patient before and after surgery, in the training set (left, *n* = 126) and test set (right, *n* = 44), respectively. Statistical significance was determined by the Mann–Whitney U test. ***p < 0.0001

Surgery is the primary treatment for breast cancer patients. Thus, we also collected post-surgery breast cancer serum samples. In the training set, there are 126 paired pre-and post-surgery breast cancer serum samples. The median level of CPT1A in serum from breast cancer patients after surgery was 32.16 ± 22.54 ng/mL, which was significantly lower than its level before surgery (Fig. 6a, b, left; 45.61 ± 45.89 ng/mL, *p* < 0.0001). In the test set, there are 44 paired pre-and post-surgery breast cancer serum samples. CPT1A levels (23.95 ± 12.70 ng/mL) consistently decreased in post-surgery serum samples of breast cancer patients compared with paired pre-surgery samples (Fig. 6a, b, right; 41.40 ± 35.02 ng/mL, *p* < 0.0001). These results indicated that CPT1A levels are positively associated with tumor burden and could serve as an indicator for disease monitoring after tumor resection.

## Discussion

Early diagnosis has significant implications for the treatment of breast cancer patients and is associated with favorable prognosis. Although mammography is the most commonly used screening method for breast cancer, it has limitations, including false-positive results and overdiagnosis. Currently, serum tumor markers such as CA15–3, CEA, and CA125 have been developed as non-invasive tools for the detection and prediction of outcomes in breast cancer [16, 35, 36]. However, less than 50% of breast cancer patients are detected with the elevation of serum CA15–3 and CEA levels, and CA125 is used mainly for detection of ovarian cancer, which is less specific in other cancer types [37, 38]. An ideal serologic marker for breast cancer is expected to serve as a diagnostic and prognostic tool with high sensitivity and specificity.

In the present study, we investigated the potential of CPT1A to serve as a diagnostic biomarker for breast cancer. Recently CPT1A has been considered as a key regulator of cancer metabolism reprogramming [39]. It was reported to be upregulated and associated with poor prognosis in several cancers, including breast cancer [20, 25, 39, 40]. According to previous studies, CPT1A amplification was found in 20% of ER-positive breast cancer cases, and the CPT1A protein level is elevated in most breast cancer cell lines [41]. CPT1A is regulated by c-MYC or AMPK in breast cancer, and promotes breast cancer metastasis or therapeutic resistance through several oncogenic signaling pathways, such as VEGF, ERK and Src pathways [25–27, 29, 42].

Although CPT1A is a mitochondrial protein, it was identified in exosomes of breast cancer cell lines in a study exploring the proteomic profile of extracellular vesicles derived from 60 cell lines of the National Cancer Institute (NCI-60) [30]. In our study, we found that CPT1A is overexpressed in breast cancer and can be detected in the cell culture medium. However, the expression patterns of intracellular and secreted CPT1A are not consistent among a panel of breast cancer cell lines. The TNBC cell lines have the highest CPT1A levels in cell culture medium, but not in cytoplasm. As we known, the generation and transport of extracellular vesicles are complex processes, which need the collaboration of sorting machineries, membrane-trafficking processes and vesicle-associated proteins [43]. Our previous study showed that CPT1A binds to several vesicular trafficking proteins in therapeutic resistant cancer cell lines, but not in therapeutic responsive cancer cell lines [22], which indicated that the vesicle-associated proteins and their interaction with CPT1A could also contribute to the extracellular level of CPT1A. Thus, the vesicular trafficking proteins in TNBC cells may promote the secretion of CPT1A. Above all, these different lines of evidence led to our investigation of CPT1A as a possible serum biomarker for breast cancer.

In this study, we found that the serum CPT1A level was significantly elevated in 430 breast cancer patients compared with either 200 patients with benign breast disease or 400 healthy controls. Several benign breast diseases, such as atypical hyperplasia, are regarded as premalignant lesions [44]. Compared with healthy controls, serum CPT1A level was elevated in patients with benign breast diseases, which indicated that CPT1A might play a significant role in the tumorigenesis and early development of breast cancer. These results further suggest that serum CPT1A could be used as a biomarker in the screening and early detection of breast cancer.

In addition, we found that serum CPT1A levels were positively correlated with metastasis, advanced TNM stage and histological grade, and may enable discrimination between different breast cancer molecular subtypes. Tumor heterogeneity has led to breast cancer classification into four intrinsic subtypes, luminal A, luminal B, HER2-enriched, and basal-like breast cancer, and these are closely related to the development and prognosis of breast cancer [45–48]. The basal-like subtype, also known as TNBC, is characterized by a lack of estrogen receptor, progesterone receptor, and HER2 expression. It comprises 10–15% of all breast cancers and leads to worse clinical outcomes than other subtypes [49–51]. According to our study, patients with TNBC exhibited significantly higher serum CPT1A levels than those with other subtypes, and CPT1A exhibited significantly high efficacy in differentiating TNBC patients from healthy controls. These results indicate that CPT1A might be a useful diagnostic and serum biomarker for breast cancer, especially for TNBC cases.

To determine the diagnostic efficacy of CPT1A, we performed ROC curve analysis and compared the AUC, sensitivity, and specificity of CPT1A and three conventional clinically used biomarkers, CA15–3, CEA, and CA125, in breast cancer samples and healthy subjects. Analysis showed that serum CPT1A displayed a higher AUC in differentiating breast cancer patients from healthy controls, with a higher and more balanced sensitivity and specificity than the other three tumor markers. Our study has identified a novel biomarker that provides a remarkable improvement in distinguishing breast cancer patients from healthy controls.

By performing a comparison of CPT1A levels in 170 paired serum samples obtained before and after surgery, we found that serum CPT1A levels decreased in breast cancer patients after surgical removal of the tumor. Thus, the serum CPT1A level was found to be an effective indicator for evaluating the surgical outcome or tumor recurrence of breast cancer patients.

Although abnormal lipid turnover has been accepted as a vital mechanism in breast cancer progress, studies investigating lipids, including TG, TC, and HDL-C, in relation to breast cancer risk have shown conflicting results [52, 53]. In our study, univariate logistic regression analysis showed that none of the lipids TG, TC, HDL-C, LDL-C, or NEFA was an effective diagnostic indicator in breast cancer patients overall, and serum CPT1A showed significant effectiveness in breast cancer diagnosis independently of these lipids.

In our study, all the participants originated from China. Although breast tumors in Asian and Caucasian women were reported to share similar molecular and genetic characteristics [54], further studies in different populations are needed. Although the CPT1A levels in breast cancer patients and healthy controls were significantly different in our study, some overlap was still observed in patients and controls (Fig. 2). Nevertheless, CPT1A could be used as an adjunct biomarker for breast cancer diagnosis. It is also necessary to determine whether serum CPT1A is a specific biomarker of breast cancer only or is also an accurate biomarker for other cancer types.

## Conclusions

Our large-scale study provided initial data about the clinically diagnostic relevance of CPT1A as a serum marker for breast cancer in both a training set and a test set. Our study showed that CPT1A could be released into cell culture medium by breast cancer cells, and serum CPT1A could be employed to distinguish breast cancer patients from healthy controls and assist in the diagnosis of breast cancer. It is positively associated with tumor burden and could serve as an indicator for disease monitoring after tumor resection. Serum CPT1A displayed a remarkably high diagnostic efficiency for breast cancer and could be a novel biomarker for the diagnosis of breast cancer.

## Supplementary Information

**Additional file 1: Table S1.** Univariate logistic regression analysis of serum CPT1A and lipids in differentiating breast cancer and controls. **Table S2.** Formulas of logistic regression models built based on the data in the training set. **Table S3.** The reference ranges of serum tumor markers and lipids. **Figure S1.** Merged original blot showing CPT1A and β-Actin bands with protein markers in a panel of non-malignant cell lines and breast cancer cell lines. β-Actin was used as a control to confirm equal loading of protein. **Figure S2.** Subject inclusion and study profile. **Figure S3.** Serum CPT1A levels in different pathologic types of breast cancer. Serum CPT1A levels of breast cancer patients with cancer in situ, invasive ductal carcinoma, invasive lobular carcinoma, invasive papillary carcinoma, mucinous carcinoma and other types, in the training set (left) and test set (right), respectively. Statistical significance was determined by the Mann–Whitney U test. **p < 0.001. **Figure S4.** The correlation of CPT1A levels and lipids concentrations in serum of breast cancer patients. Co-expression analysis of CPT1A versus TG (A), TC (B), HDL-C (C), LDL-C (D) and NEFA (E) levels in serum of breast cancer patients in the training set (left) and test set (right), respectively. The Spearman’s correlation coefficient was calculated using the GraphPad software program. **Figure S5.** The ROC curve analyses of CPT1A, CA15–3, CEA and CA125 in the differentiation of breast cancer cases from healthy controls by using 10-fold cross validation in training set. 10-fold cross validated ROC curves of CPT1A (A), CA15–3 (B), CEA (C), CA125 (D), the combination of CA 15–3, CEA and CA 125 (E), and the combination of the four markers (F).

## Data Availability

The datasets used and/or analysed during the current study are available from the corresponding author on reasonable request.

## Abbreviations

AMPK: Adenosine 5′-monophosphate-activated protein kinase
CA15–3: Cancer antigen 15–3
CEA: Carcinoembryonic antigen
CPT1A: Carnitine palmitoyl transferase 1A
AUC: Area under the curve
ERK: Extracellular regulated protein kinase
EVs: Extracellular vesicles
FAO: Fatty acid oxidation
HER2: Human epidermal growth factor receptor 2
HDL-C: High-density lipoprotein-cholesterol
IQR: Interquartile range
LDL-C: Low-density lipoprotein-cholesterol
NEFA: Non-esterified fatty acids
NPV: Negative predictive value
PPV: Interquartile range
ROC: Receiver operating characteristic
TC: Total cholesterol
TNBC: Triple-negative breast cancer
TNM: Tumor node metastasis
TG: Triglyceride
-LR: Negative likelihood ratio
+LR: Positive likelihood ratio
