# Correction: Carnitine palmitoyl transferase 1 A is a novel diagnostic and predictive biomarker for breast cancer

**DOI:** 10.1186/s12885-023-10518-w

**Published:** 2023-01-11

**Authors:** Zheqiong Tan, Yaru Zou, Man Zhu, Zhenzhao Luo, Tangwei Wu, Chao Zheng, Aqing Xie, Hui Wang, Shiqiang Fang, Shuiyi Liu, Yong Li, Zhongxin Lu

**Affiliations:** 1 Department of Medical Laboratory, the Central Hospital of Wuhan, Tongji Medical College, Huazhong University of Science and Technology, Wuhan, 430014 Hubei China; 2 Department of Clinical Laboratory, Wusong Central Hospital, Baoshan District, Shanghai, 200940 China; 3 Cancer Research Institute of Wuhan, Wuhan, 430014 Hubei China; 4 Department of Central Laboratory, the Central Hospital of Wuhan, Tongji Medical College, Huazhong University of Science and Technology, Wuhan, 430014 Hubei China; 5 Department of Cancer Biology, Lerner Research Institute, Cleveland Clinic, Cleveland, OH 44195 USA; 6 Department of Medicine, Section of Epidemiology and Population Sciences, Baylor College of Medicine, Houston, TX 77030 USA

**Correction: BMC Cancer 21, 409 (2021)**

**https://doi.org/10.1186/s12885-021-08134-7**

Following publication of the original article [1], the authors reported an error in the funding section. The funding number for the National Science Foundation of China was incorrect. The funding statement should read as follows:

This research was funded by National Science Foundation of China (81802652), Research Fund of Hubei Province Health Bureau (WJ2015MB144) and Research Fund of Wuhan Public Health Bureau (WX15A12 and WX18Y11). The funders had no role in the design of the study, data collection, analysis, interpretation of data, or in writing the manuscript.
