# Supplementary material for: Carnitine palmitoyl transferase 1A is a novel diagnostic and predictive biomarker for breast cancer
Source: BMC Cancer. 2021 Apr 15;21:409. doi: 10.1186/s12885-021-08134-7 (PMC8048260; doi:10.1186/s12885-021-08134-7)
Supplement: Supplementary file 1 — Additional file 1: Table S1. Univariate logistic regression analysis of serum CPT1A and lipids in differentiating breast cancer and controls. Table S2. Formulas of logistic regression models built based on the data in the training set. Table S3. The reference ranges of serum tumor markers and lipids. Figure S1. Merged original blot showing CPT1A and β-Actin bands with protein markers in a panel of non-malignant cell lines and breast cancer cell lines. β-Actin was used as a control to confirm equal loading of protein. Figure S2. Subject inclusion and study profile. Figure S3. Serum CPT1A levels in different pathologic types of breast cancer. Serum CPT1A levels of breast cancer patients with cancer in situ, invasive ductal carcinoma, invasive lobular carcinoma, invasive papillary carcinoma, mucinous carcinoma and other types, in the training set (left) and test set (right), respectively. Statistical significance was determined by the Mann–Whitney U test. **p < 0.001. Figure S4. The correlation of CPT1A levels and lipids concentrations in serum of breast cancer patients. Co-expression analysis of CPT1A versus TG (A), TC (B), HDL-C (C), LDL-C (D) and NEFA (E) levels in serum of breast cancer patients in the training set (left) and test set (right), respectively. The Spearman’s correlation coefficient was calculated using the GraphPad software program. Figure S5. The ROC curve analyses of CPT1A, CA15–3, CEA and CA125 in the differentiation of breast cancer cases from healthy controls by using 10-fold cross validation in training set. 10-fold cross validated ROC curves of CPT1A (A), CA15–3 (B), CEA (C), CA125 (D), the combination of CA 15–3, CEA and CA 125 (E), and the combination of the four markers (F). [file 12885_2021_8134_MOESM1_ESM.docx]

Table S1. Univariate logistic regression analysis of serum CPT1A and lipids in differentiating breast cancer and controls

|  | **Training set** | | |  | **Test set** | | |
| --- | --- | --- | --- | --- | --- | --- | --- |
| Variable | OR^a^ | 95% CI^b^ | *p* Value |  | OR^a^ | 95% CI^b^ | *p* Value |
| CPT1A | 1.124 | 1.100-1.148 | 0.000 |  | 1.092 | 1.062-1.124 | 0.000 |
| TG | 0.914 | 0.776-1.076 | 0.280 |  | 0.991 | 0.965-1.018 | 0.524 |
| TC | 1.021 | 0.953-1.094 | 0.585 |  | 1.005 | 0.943-1.070 | 0.886 |
| HDL-C | 0.982 | 0.923-1.044 | 0.560 |  | 0.589 | 0.283-1.227 | 0.158 |
| LDL-C | 1.002 | 0.957-1.048 | 0.942 |  | 0.993 | 0.946-1.043 | 0.779 |
| NEFA | 0.930 | 0.793-1.091 | 0.373 |  | 0.953 | 0.842-1.098 | 0.623 |

Abbreviations: TG: triglyceride; TC: total cholesterol; HDL-C: high-density lipoprotein- cholesterol; LDL-C: low-density lipoprotein-cholesterol; NEFA: non-esterified fatty acids.

^a^Odds ratio (OR) estimated from logistic regression model; ^b^Confidence interval (CI) of estimated OR.

Table S2. Formulas of logistic regression models built based on the data in the training set.

|  | **Logistic regression models** |
| --- | --- |
| Breast cancer VS. Controls |  |
| CPT1A | Logit (P) = -3.312 + 0.125 (CPT1A) |
| CA153 | Logit (P) = -0.304 + 0.036 (CA153) |
| CEA | Logit (P) = -0.445 + 0.246 (CEA) |
| CA125 | Logit (P) = -0.108 + 0.12 (CA125) |
| CA153+CEA+CA125 | Logit (P) = -0.768 + 0.019 (CA153) + 0.231 (CEA) + 0.010 (CA125) |
| CPT1A+CA153+CEA+CA125 | Logit (P) = -3.920 + 0.125 (CPT1A) - 0.008 (CA153) + 0.235 (CEA) + 0.013 (CA125) |
| Breast cancer VS. Benign breast disease |  |
| CPT1A | Logit (P) = -1.76 + 0.061 (CPT1A) |
| TNBC VS. Controls |  |
| CPT1A | Logit (P) = -5.983 + 0.141 (CPT1A) |

Table S3. The reference ranges of serum tumor markers and lipids.

|  | **Reference ranges** |
| --- | --- |
| CA15-3 | <31.3 U/mL |
| CEA | <5.0 ng/mL |
| CA125 | <35 U/mL |
| TG | <1.7 mmol/L |
| TC | <5.18 mmol/L |
| HDL-C | >1.04 mmol/L |
| LDL-C | <3.37 mmol/L |
| NEFA | 0.172–0.586 mmol/L |

Abbreviations: CA15-3: cancer antigen 15-3; CEA: carcinoembryonic antigen; CA-125: cancer antigen 125; TG: triglyceride; TC: total cholesterol; HDL-C: high-density lipoprotein- cholesterol; LDL-C: low-density lipoprotein-cholesterol; NEFA: non-esterified fatty acids.

**Supplementary figures**

**
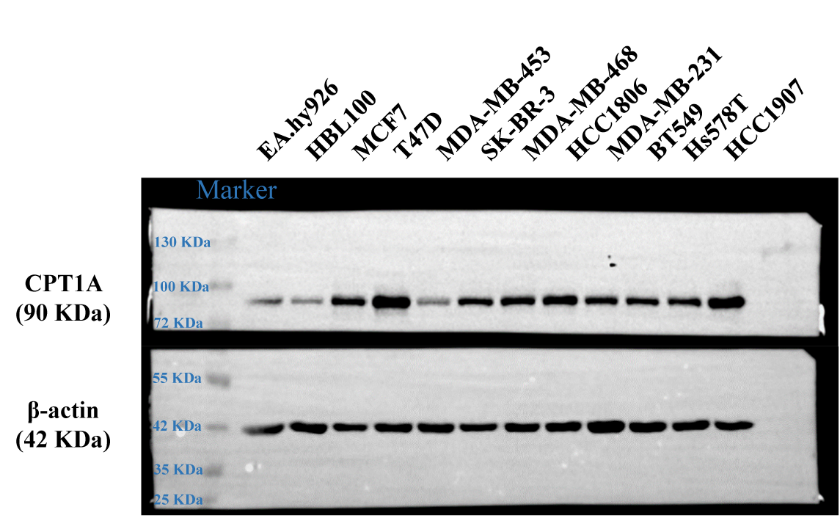
**

Figure S1. Merged original blot showing CPT1A and β-Actin bands with protein markers in a panel of non-malignant cell lines and breast cancer cell lines. β-Actin was used as a control to confirm equal loading of protein.


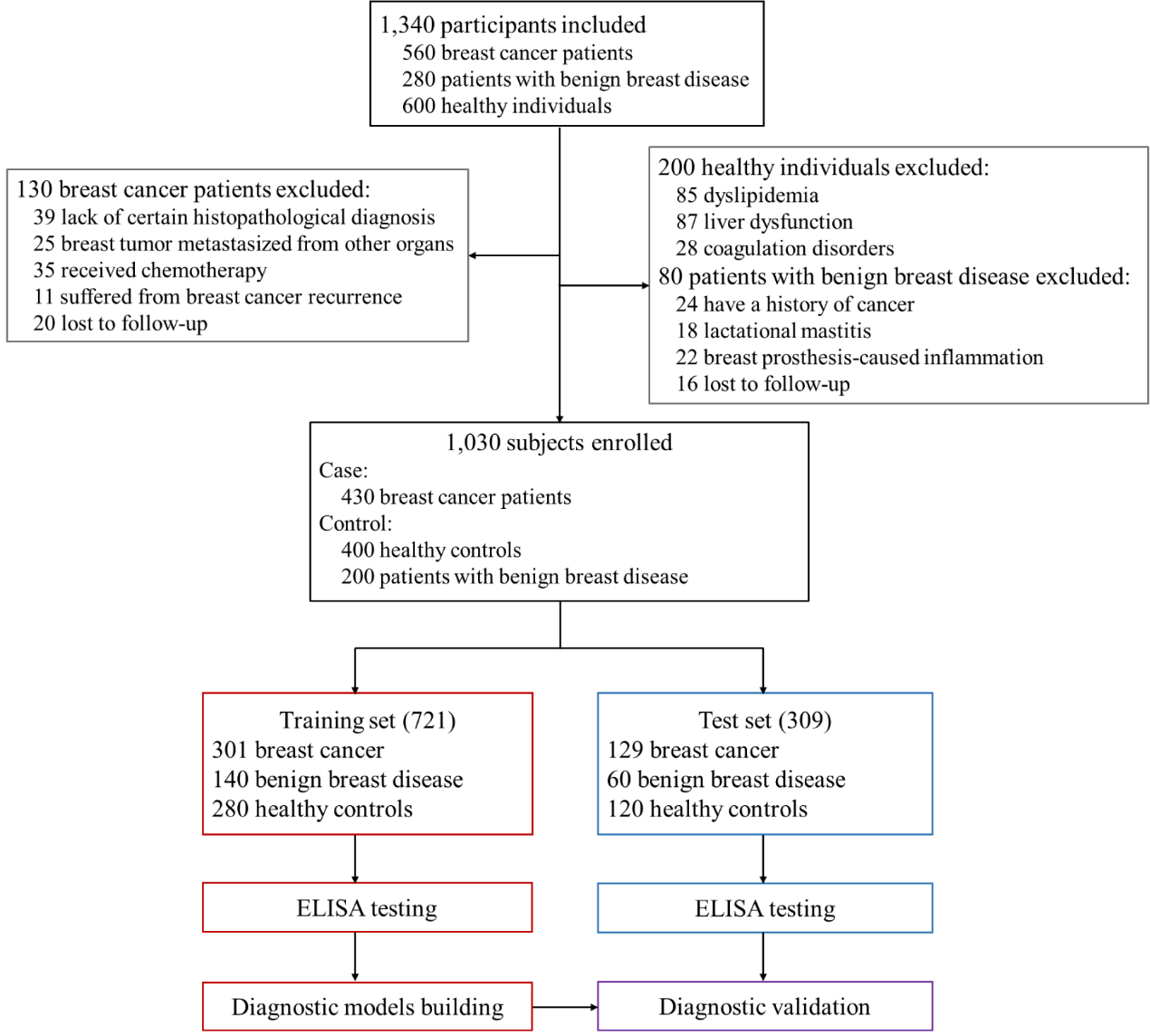


Figure S2. Subject inclusion and study profile.


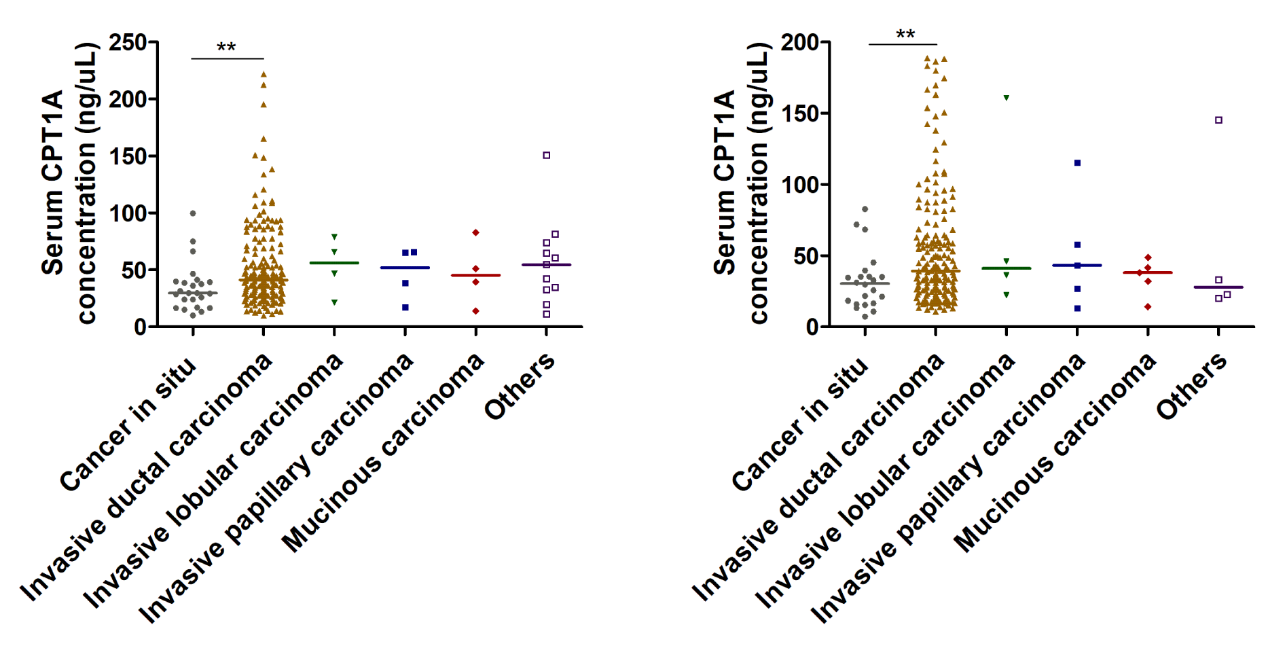


Figure S3. Serum CPT1A levels in different pathologic types of breast cancer. Serum CPT1A levels of breast cancer patients with cancer in situ, invasive ductal carcinoma, invasive lobular carcinoma, invasive papillary carcinoma, mucinous carcinoma and other types, in the training set (left) and test set (right), respectively. Statistical significance was determined by the Mann–Whitney U test. ***p*<0.001.


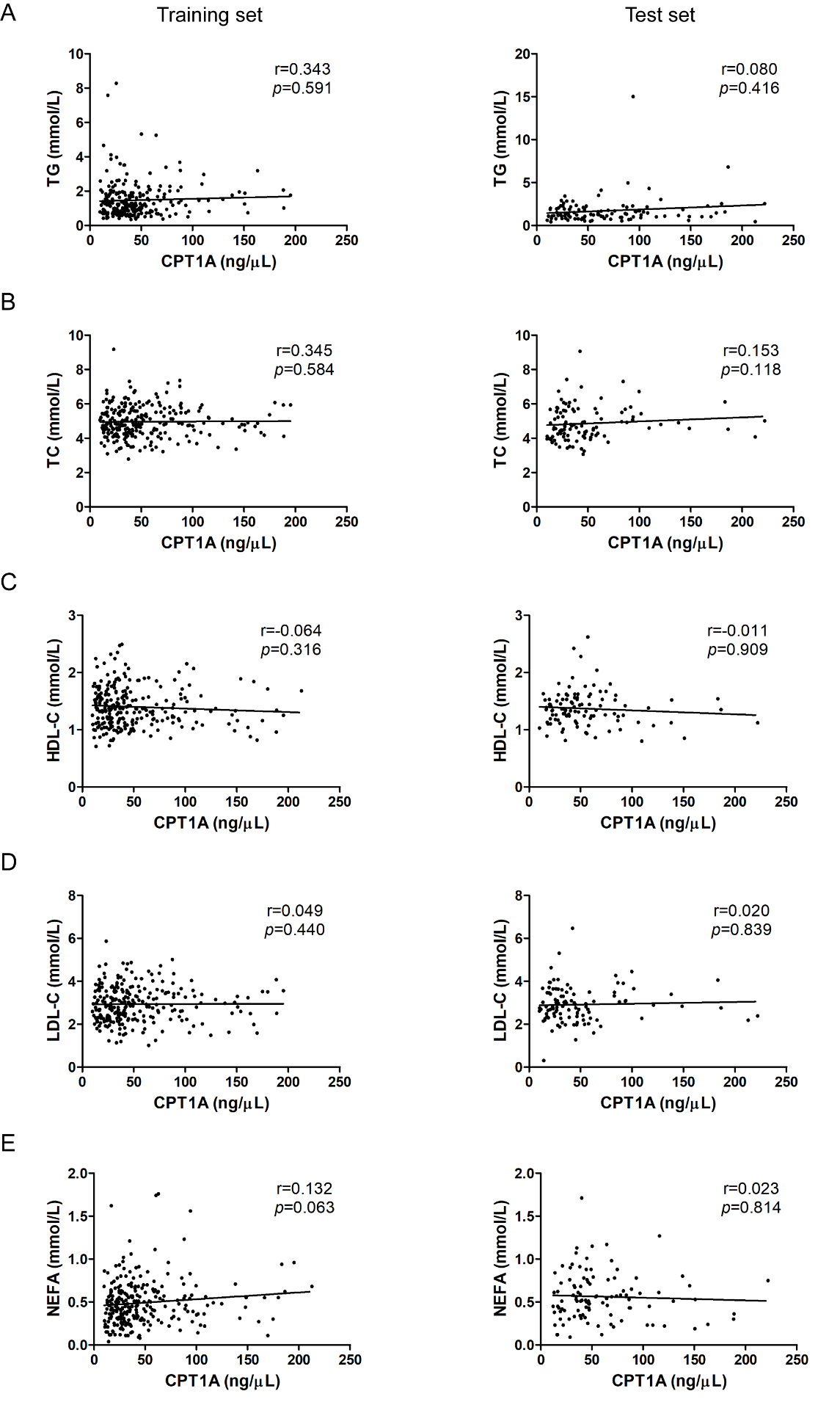
Figure S4. The correlation of CPT1A levels and lipids concentrations in serum of breast cancer patients. Co-expression analysis of CPT1A versus TG (A), TC (B), HDL-C (C), LDL-C (D) and NEFA (E) levels in serum of breast cancer patients in the training set (left) and test set (right), respectively. The Spearman’s correlation coefficient was calculated using the GraphPad software program.


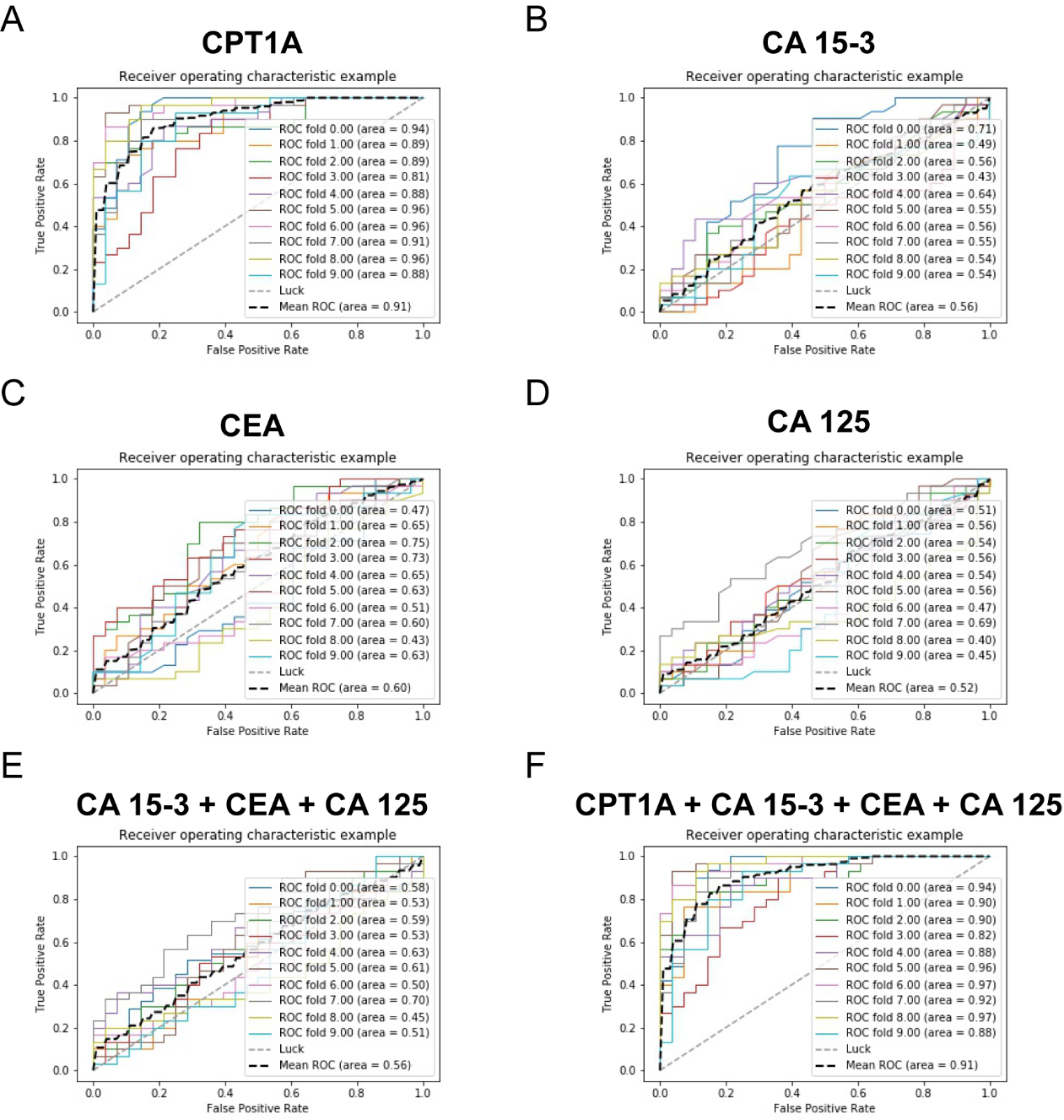
Figure S5. The ROC curve analyses of CPT1A, CA15-3, CEA and CA125 in the differentiation of breast cancer cases from healthy controls by using 10-fold cross validation in training set. 10-fold cross validated ROC curves of CPT1A (A), CA15-3 (B), CEA (C), CA125 (D), the combination of CA 15-3, CEA and CA 125 (E), and the combination of the four markers (F).
